# Antimicrobial and antibiofilm effects of cyclic dipeptide-rich fraction from *Lactobacillus plantarum* loaded on graphene oxide nanosheets

**DOI:** 10.3389/fmicb.2024.1391039

**Published:** 2024-09-02

**Authors:** Farid Shirmardi Dezaki, Tahmineh Narimani, Mustafa Ghanadian, Elham Bidram, Farkhondeh Poursina

**Affiliations:** ^1^Department of Microbiology, School of Medicine, Isfahan University of Medical Sciences, Isfahan, Iran; ^2^Isfahan Pharmaceutical Sciences Research Center, Isfahan University of Medical Sciences, Isfahan, Iran; ^3^Biosensor Research Center (BRC), Department of Biomaterials, Nanotechnology, and Tissue Engineering, School of Advanced Technologies in Medicine, Isfahan University of Medical Sciences (IUMS), Isfahan, Iran

**Keywords:** *Staphylococcus aureus*, cyclodipeptide, graphene oxide, *Acinetobacter baumannii*, antimicrobial, antibiofilm, nanosheets

## Abstract

**Introduction:**

One effective method to combat bacterial infections is by using bacteria itself as a weapon. *Lactobacillus* is a type of fermenting bacterium that has probiotic properties and has demonstrated antimicrobial benefits against other bacteria. Cyclodipeptides (CDPs), present in the supernatant of *Lactobacillus*, possess several antimicrobial properties.

**Methods:**

In this study, the CDP fraction was isolated from the supernatant of *Lactobacillus plantarum* (*L. plantarum*). This fraction was then loaded onto graphene oxide nanosheets (GO NSs). The study assessed the substance’s ability to inhibit bacterial growth by using the minimum inhibitory concentration (MIC) method on *A. baumannii* and *S. aureus* strains that were obtained from clinical samples. To determine the substance’s impact on biofilm formation, the microtiter plate method was used. Moreover, the checkerboard technique was employed to explore the potential synergistic effects of these two substances.

**Results and discussion:**

According to the study, the minimum inhibitory concentration (MIC) of the desired compound was found to be 1.25 mg/mL against *S. aureus* and 2.5 mg/mL against *A. baumannii*. Furthermore, at a concentration of 10 mg/mL, the compound prevented 81.6% (*p* < 0.01) of biofilm production in *A. baumannii*, while at a concentration of 1.25 mg/mL, it prevented 47.5% (*p* < 0.05) of biofilm production in *S. aureus*. The study also explored the synergistic properties of two compounds using the checkerboard method.

**Conclusion:**

In general, we found that GO NSs possess antimicrobial properties and enhance cyclodipeptides’ activity against *S. aureus* and *A. baumannii*.

## 1 Introduction

Bacteria and fungi can be inhibited by lactic acid bacteria (LAB) and their cultured supernatants (Rouse and van Sinderen, 2008). The primary cause of the antibacterial effect of LAB is the generation of lactic and acetic acids. Bacteriocins, which are also important, are synthesized by certain strains (Lindgren and Dobrogosz, 1990; Vandenbergh, 1993). During metabolic processes in bacterial cultures, *Lactobacillus* spp. produces antimicrobial substances that inhibit Gram-positive and negative bacteria and fungi. These substances contain different CDPs and have been extensively examined (Dec et al., 2014; Hedberg et al., 2008; Borthwick and Da Costa, 2017). CDPs are desirable scaffolds for drug design due to the structural diversity of their chiral side chains (Lee et al., 2010). Graphene is the thinnest material known to us. It is created by chemically exfoliating graphite. Graphene oxide (GO) has different functional groups, including hydroxyl, carbonyl, epoxy, and carboxylic groups. These groups make GO soluble in water and potentially useful in human therapeutics (Loh et al., 2010). Bioscientists have taken an interest in a flat sheet of carbon that exists in two dimensions. This is because it has unique and useful physical and chemical properties. One of its notable features is its high surface-to-volume ratio, and it is also better suited to living organisms than other materials (Lok et al., 2006; Ahamed et al., 2010). GO has some great properties that make it useful for delivering cancer drugs. It has a large surface area, can hold a lot of drugs, and reacts to changes in pH. Some scientists think that GO could be used to carry drugs like doxorubicin, camptothecin, and methotrexate (Wang et al., 2018; Gong et al., 2017; Tu et al., 2017; Liu et al., 2008; Miao et al., 2013). Hospital-acquired bacterial infections can lead to problems in treatment and even death due to antibiotic resistance. *A. baumannii* is responsible for several illnesses, such as bloodstream infections, pneumonia from ventilators, meningitis, urinary tract infections, heart inflammation, and wound infections (Gordon and Wareham, 2010). *A. baumannii* is a type of harmful bacteria that can cause infections in hospitals. These infections can be very serious and even lead to death (Bergogne-Berezin and Towner, 1996). *A. baumannii* is a type of bacteria that is resistant to many drugs, making it hard to treat. Because of this, it is considered one of the top seven pathogens that pose a threat to healthcare delivery systems (Talbot et al., 2006). Bacteria can survive in the presence of antibiotics by forming biofilms, especially *A. baumannii* and *S. aureus*. This types of bacteria are a common cause of biofilm-related contamination in medical devices (Singhai et al., 2012). *S. aureus* is a type of bacteria that can cause a range of infections, from minor skin infections to post-operative wound infections. This Gram-positive bacterium is known to be a potential pathogen (Skinner and Keefer, 1941). Our study aims to explore the effects of *L. plantarum* CDPs loaded on GO NSs on resistant *A. baumannii* and *S. aureus* isolated from clinical samples.

## 2 Materials and methods

### 2.1 Materials

The standard strains of *L. plantarum* (ATCC 14917), *S. aureus* (ATTC 25923), and *A. baumannii* (ATCC 19606) were purchased from the Iranian Biological Resource Center, De Man, Rogosa, and Sharpe (MRS) medium were purchased from HIMEDIA (India), Mueller Hinton Broth (MH) were purchased from HIMEDIA (India), GO sheets were purchased from Sigma-Aldrich (America), Amberlite IRA410 ion exchanger resin was purchased from Chemika (Germany); dichloromethane solvent was purchased from Merck (Germany), glass column was purchased from Agilent (USA).

### 2.2 Fractionation of antimicrobial substances

To prepare *L. plantarum* supernatant, the first step was to culture the bacterium in a liquid MRS medium (2 Liters). The culture was then incubated in microaerophilic conditions at a temperature of 37°C for 72 h. After this, the culture medium containing the grown bacteria was centrifuged at 4000 rpm for 20 min and the supernatant was separated (Lin et al., 2015). This solution contains peptide compounds along with nutrients inside the culture medium and possibly other metabolites secreted by *L. plantarum* in the culture medium. To separate CDPs according to their solubility in organic solvent, the solvent-solvent extraction method was used in the separating funnel. In the first step, 2 liters of supernatant were poured into an Erlenmeyer flask, and the pH of the solution was adjusted to 9 with the assistance of ammonia. The initial pH of the solution was 5.5. 400 mL of the supernatant was successively poured into a separating funnel and 400 mL of dichloromethane solvent was added to it (in a 1:1 ratio) and vigorously stirred to separate the two phases. Finally, the dichloromethane phase was poured concentrated, and dried with a rotary device connected to a vacuum pump at a temperature of 40°C. This sample was stored in a freezer at −20°C until use. The next step was to separate CDP compounds from non-cyclic peptides and free amino acids with carboxyl groups using ion exchange resin. The characteristic of the desired CDP compounds is that they do not have a carboxyl group. For this purpose, a 20 × 200 mm glass column filled with IRA410 anion exchange resin was used (Kwak et al., 2018). First, the column was washed with 50% water–methanol and then 14% ammonia was passed through the column to activate the ions and wash. Then the concentrated solution that was extracted from the supernatant was added to the column and 50% water–methanol solution was added to the column each time so that the CDPs passed through the column. In this step, non-cyclic peptide compounds and free amino acids containing anionic carboxyl groups were absorbed into the resin, and other compounds, including cyclic dipeptides, passed through the column. In the end, the solution removed from the column was collected in glass vials with a volume of 5 mL, and the OD of all vials was read with a spectrophotometer at 280 nm. All vials with maximum absorbance at 280 nm containing CDP compounds were added together and set aside as a rich fraction of CDP compounds.

### 2.3 Nanosheets synthesis and loading CDPs on GO NSs

Due to the particular physical and chemical properties of GO, the large micro-sized flakes of this material can be converted into smaller sheets under 300 nm using water bath ultrasonication for more than 10 h. The combination of mechanical forces, chemical interactions, and surface properties under ultrasonication plays a crucial role in converting micro-GO into nanosheets (Li et al., 2008; Zhu et al., 2010). Notably, the concentration of the initial sample (4 mg/mL) required to be diluted into around 0.1 mg/mL ensuring that all sheets were accessible to the sonication waves. To prepare the mixture, 20 mg of lyophilized CDP powder was dissolved in 1 mL of deionized distilled water and mixed with a solution of GO NSs. The mixture was stirred magnetically for 12 h at a temperature of 25°C. Finally, by washing three times and using a centrifuge with a speed of 13,000 rpm, the precipitate was collected, lyophilized, and dried. To prepare the final stock of CDPs fraction composition loaded on GO NSs, 20 mg of this dry powder was dissolved in 1 mL of sterile deionized distilled water (Li et al., 2021; Mei et al., 2021).

### 2.4 Characterization techniques

To prepare the samples for the Gas Chromatography–Mass Spectrometry test (GC–MS), first, the sample was humidified and prepared in a dry and frozen form using a freeze dryer under the freezing and sublimation process. In the next step, 30 mg of dry sample was added with 50 μL of BSTFA reagent +10% TMCS with the sampler and then incubated for 5 min at 70°C to complete the derivatization process. Then, 1 μL of the sample was injected into the gas chromatography machine (Agilent Technologies 7890A, United States) with a special syringe. The identification of mass spectra was done by comparing them with the basic spectra available in the computer database of GC–MS and library sources (NIST 98 library). Also, the identification of chemical compounds was done based on the comparison of their absorption indices (Kowats index) and mass spectrum with the compounds given in the source (Hoque et al., 2010). The presence of functional groups and molecular interactions between compounds were investigated using Fourier-transform Infrared Spectroscopy (FTIR) in the wavelength range of 400–4,000 cm^−1^. The size of the nanosheets was measured with Field Emission Scanning Electron Microscopy (FE_SEM Quanta-FEG450, United States). EDAX, or X-ray Energy Diffraction Spectroscopy, is an additional feature that can be found in SEM devices. It is used to determine the elemental composition of solid samples by analyzing the unique X-ray energy they emit. This analysis allows one to examine the samples and determine the type of element present, as well as its weight or atomic percentage.

### 2.5 Patients and specimens

Clinical isolates were collected from the microbiology department of Omid Hospital in Isfahan. These clinical samples were then transferred to the Isfahan microbiology laboratory of the Faculty of Medicine for further analysis. A total of 16 clinical samples of *A. baumannii* that were resistant to antibiotics (Imipenem, Meropenem, Amikacin, Cefepime, Ciprofloxacin, Trimethoprim-sulfamethoxazole, Ceftazidime) in addition to 5 clinical samples of *S. aureus* were collected. To identify the isolates, Gram staining was done. Then, *A. baumannii* samples were placed on blood agar, Eosin Methylene Blue (EMB), and McConkey agar, and *S. aureus* samples were placed on blood agar and Mannitol Salt Agar (MSA) and incubated for 24 h. Also, biochemical tests including MRVP, TSI, OF, Simon Citrate, oxidase, and catalase tests were performed for *A. baumannii* and catalase, oxidase, DNase, and culture on mannitol salt agar for *S. aureus*.

### 2.6 Antibacterial activity

To obtain the MIC, microbial suspension was initially prepared at a concentration of 0.5 McFarland. It was then diluted with MH broth, resulting in a final concentration of 10^6^ CFU/mL. Additionally, three different stocks were prepared: GO NSs (32 mg/mL), CDP fraction stock (10 mg/mL), and CDP fraction loaded on GO NSs (20 mg/mL). The plates were then placed on a shaker for 60 min and incubated for 24 h at 37°C. The first concentration where turbidity was not observed was considered MIC, and to obtain the lethal concentration, 50 μL of each well was spread on blood agar and incubated overnight at 37°C. The first concentration in which no bacterial colony was observed was considered as MBC. Triplicate sets were maintained for each concentration of the test sample.

### 2.7 Checkerboard dilution test

Checkerboard analysis is a method used to measure the synergy or synergism of antimicrobial compounds, by comparing their potency alone and in combination. This comparison is expressed as the Fractional Inhibitory Concentration index (FIC). In this study, checkerboard assay was performed using different concentrations of CDP and GO NSs, ranging from 1/8 to 4X MIC. The CDP fraction stock had a concentration of 5 mg/mL for *S. aureus* and 10 mg/mL for *A. baumannii*, whereas the GO NSs stock had a concentration of 16 mg/mL for *S. aureus* and 32 mg/mL for *A. baumannii*. The concentrations for GO were varied from 8 mg/mL to 0.25 mg/mL for *S. aureus* and from 16 mg/mL to 0.5 mg/mL for *A. baumannii*, while the concentrations for CDP were varied from 2.5 mg/mL to 0.15 mg/mL for *S. aureus* and from 5 mg/mL to 0.31 mg/mL for *A. baumannii*. Following the addition of 150 μL of MH broth to each of the 96-well plates, 5 μL of microbial bacterial suspension (0.5 McFarland) was added. The final concentration of bacteria in each well was 10^6^ CFU/mL. The CDP dilutions were prepared horizontally across the plate, while the GO NSs dilutions were arranged vertically from the top to the bottom rows of the plate. All plates were incubated at 37°C for 24 h. The FIC index was determined for each compound in every combination by using the following formula: FICA + FICB = FICS, where FICA equals the MIC of drug A in combination divided by the MIC of drug A alone and FICB equals the MIC of drug B in combination divided by the MIC of drug B alone (as shown in Equations 1, 2) (Lee et al., 2012; Mazur et al., 2020).


(1) $${FIC}_{A}=\frac{\mathrm{MIC}\;\mathrm{of}\;\mathrm{compound}\;A\;\mathrm{in}\;\mathrm{combination}}{\mathrm{MIC}\;\mathrm{of}\;\mathrm{compound}\;A\;\mathrm{alone}}$$

(2) $${FIC}_{B}=\frac{\mathrm{MIC}\;\mathrm{of}\;\mathrm{compound}\;B\;\mathrm{in}\;\mathrm{combination}}{\mathrm{MIC}\;\mathrm{of}\;\mathrm{compound}\;B\;\mathrm{alone}}$$

### 2.8 Antibiofilm activities

The effectiveness of the CDP fraction loaded on GO NSs against biofilms was tested using the crystal violet assay. The compounds were diluted twofold from 0.01 mg/mL to 10 mg/mL in a sterile 96-well microtiter plate. To each well containing 100 μL MH broth and varying concentrations of the compound, 5 μL of an overnight incubated strains suspension (0.5 McFarland) was added. The mixture was then incubated at 37°C for 48 h. The well used for positive control consisted of MH broth and bacterial suspension that was not treated. On the other hand, the negative control well contained MH broth without compounds and bacteria. After the incubation period, the contents of the wells were removed and washed. This was done by adding 200 μL of 96% ethanol for 15 min and then it was placed at 37°C for 30 min until dry. The wells were stained using 200 μL of 1% (w/v) crystal violet for 15 min to visualize the biofilms formed and washed with deionized water five times. To determine the optical densities of the stained adherent bacteria, a mixture of 33% acetone and 80% ethanol in a 1:1 ratio was added to the plate. An ELISA microplate reader was used to measure the absorbance at 595 nm, which was recorded for further analysis. The antibiofilm activity ratio of the compounds was calculated using Equation 3.


(3) $$\begin{array}{l} \%\mathrm{preventing}\;\mathrm{biofilm}\;\mathrm{formation}=\\ 100-\;\frac{OD\;\mathrm{tested}\;\mathrm{compounds}}{OD\;\mathrm{positive}\;\mathrm{control}}\times 100 \end{array}$$

### 2.9 Statistical analysis

Data were expressed as means ± standard deviation (SD) of at least three independent experiments. To compare the prevention of biofilm formation in bacteria with the positive control, data were compared for statistical significance using one-way analysis of variance (ANOVA) followed by Tukey’s test in SPSS software (version 23) and GraphPad Prism software (version 8.0.2). The level of significance was set at *p* < 0.05, and *p* < 0.01 was considered extremely significant.

## 3 Results and discussion

### 3.1 Column preparation and CDPs fraction

**Figure 1:**
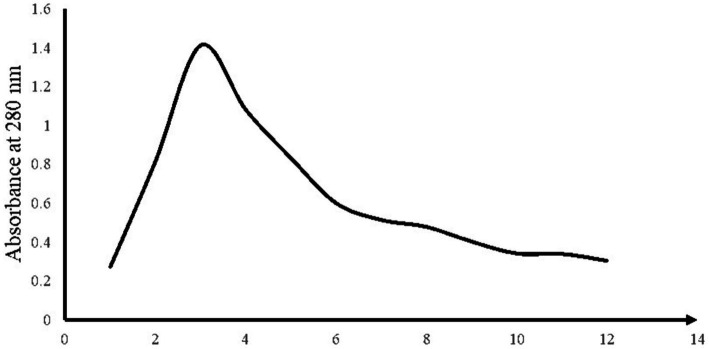
OD_280_ results of different injections into the column.

After preparing the column and filling it with IRA410 ion exchange resin, the column output was collected in glass vials. The ODs of all vials were read with a spectrophotometer at a wavelength of 280 nm, and the results are shown in Figure 1. All vials with the highest absorbance at 280 nm were selected as the fraction containing CDP compounds.

### 3.2 Characteristics of nanosheets and compounds

**Figure 2:**
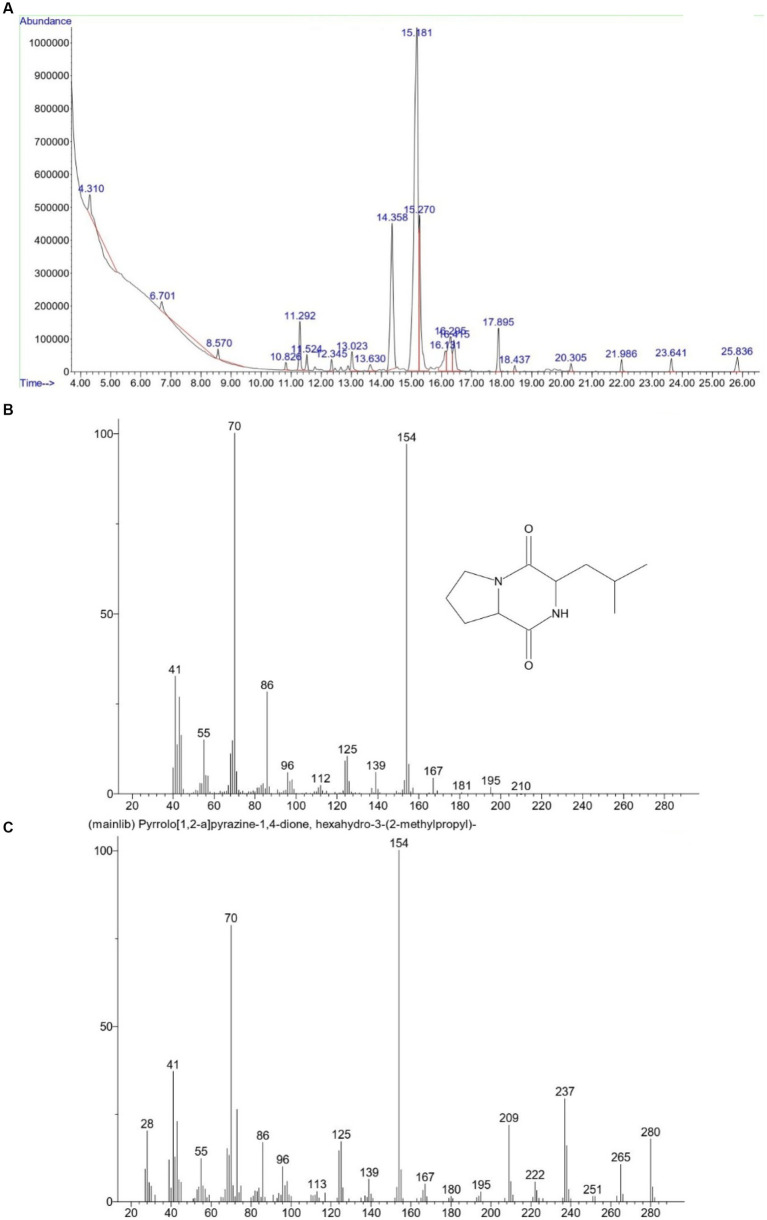
Mass spectrometer analysis of the compound isolated in 16.299 min **(A)** and comparing its spectrum **(B)** with the spectrum of the standard CDP structure **(C)**.

**Figure 3:**
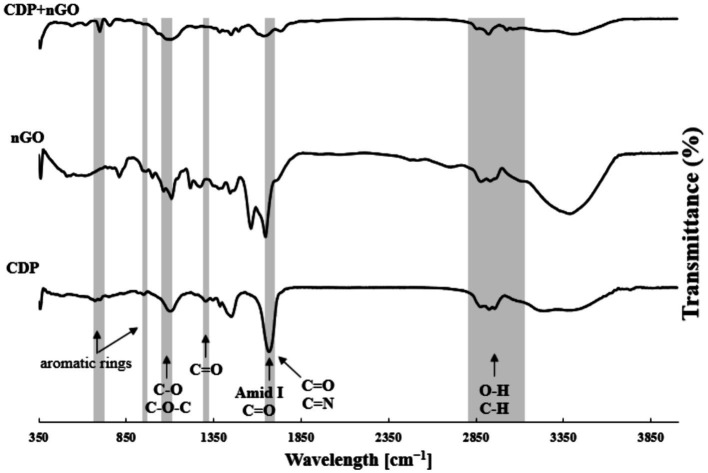
The FTIR spectrum of compounds in the CDP, GO NSs and CDP loaded on GO NSs.

**Figure 4:**
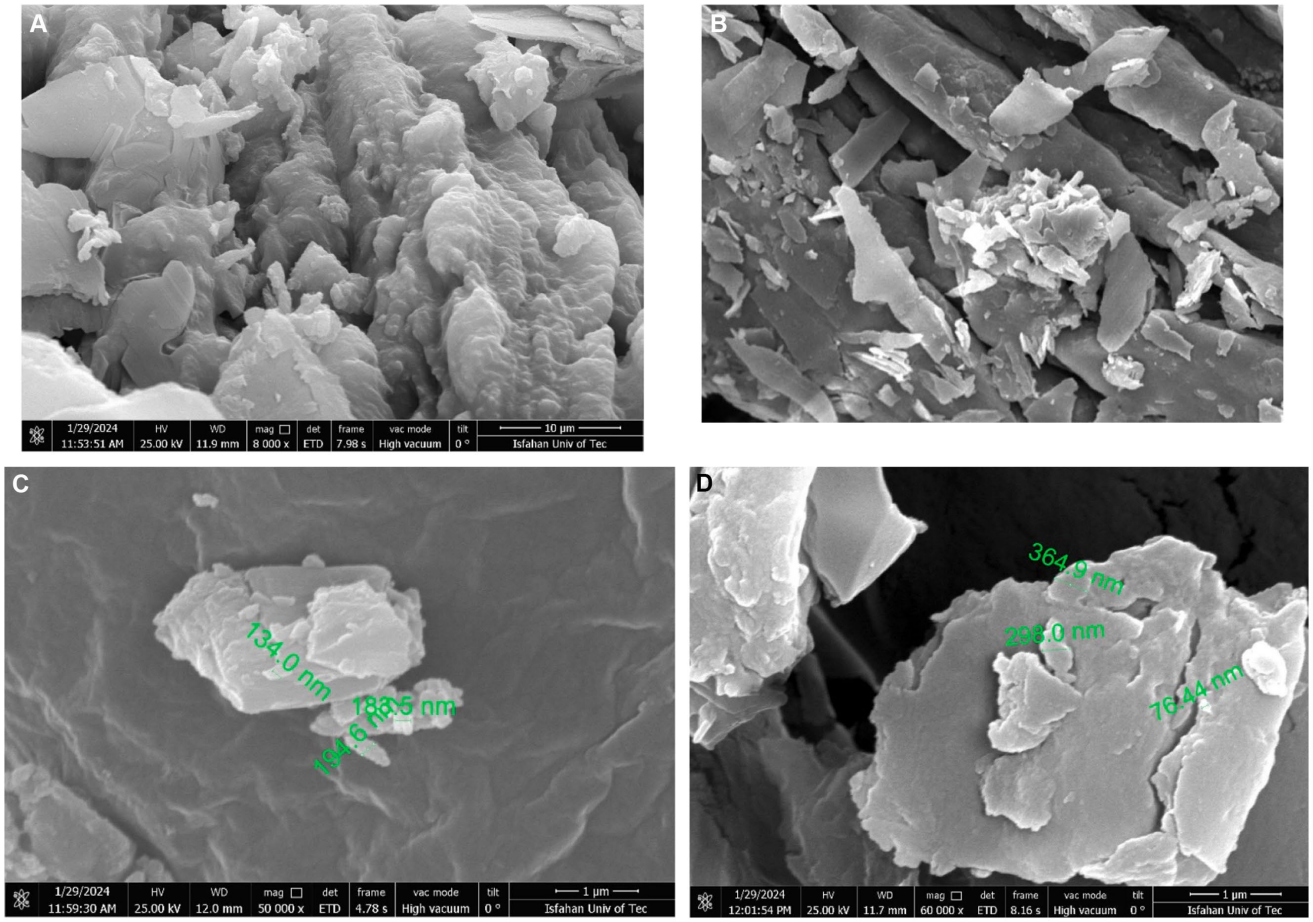
FE-SEM analysis.

GC–MS analysis was performed from the vials with the highest absorbance at 280 nm. Results are shown in Figure 2. By examining the GC–MS analysis data and the data stored in the library, it was concluded that the desired compound, Pyrrolo[1,2-a]pyrazine-1,4-dione, hexahydro-3-(2-methylpropyl), existed and was isolated in the separated fractions at 16.299 min. In a study conducted in 2018 by Kwak et al., the CDP fraction separation method was through a Semi-preparative HPLC System. Similar to our study, dichloromethane solvent, and amberlite IRA-67 ion exchange resin were used to remove organic acids and sugars. Then, 17 different fractions were obtained, each containing different CDPs. The GC–MS results of their study showed that the supernatant of *L. plantarum* had 16 different types of CDPs, the use of which in combination had significant antiviral and antifungal properties (Kwak et al., 2018). Infrared spectroscopy was performed to measure and detect the compounds in the CDPs fraction and it is shown in Figure 3. In the range of 1,050 cm^−1^ to 1,100 cm^−1^, there are peaks of functional groups C-O and C-O-C. The strong peak in the region of 1,650 cm^−1^ indicates the C=O functional group and also indicates the presence of the amide I bond in the CDPs fraction. The peaks in the range of 2,800 cm^−1^ to 3,500 cm^−1^ are related to O-H and C-H bonds, which have decreased in the spectrum of the loaded compound, which indicates the involvement of these functional groups in hydrogen bonds. The peaks at 1666 cm^−1^ to 1,685 cm^−1^ correspond to C=O and C=N, the peak at 1302 cm^−1^ corresponds to the C=O group, the peaks at 669 cm^−1^, 759 cm^−1^, and 948 cm^−1^ can be due to the presence of aromatic rings in the CDPs fraction that they are consistent with previous studies (Krivorotova et al., 2016). In the spectrum of the combination of the two substances, the intensity of the high peaks decreased, which indicates that there are interactions between the CDPs fraction and the GO NSs. In addition, the peak related to C=O in the range of 1,650 cm^−1^ is due to the synergistic effect of hydrogen bonding between the CDPs fraction and oxygenated groups in GO NSs, the electrostatic interaction between the CDPs fraction, and the negative charge on the surface of the nanosheets (Suneetha, 2018). Similar findings were observed by Kumar et al. (2023), Kanchanapally et al. (2015), and Li et al. (2021) on the loading of various peptides on GO. By reviewing the articles, we found that Mei et al., published an article in 2021 that worked on loading a type of antimicrobial peptide called OH-CATH30 on pegylated GO (Mei et al., 2021). Similar to our research, the manufacturing method was as follows. GO was strongly mixed with the peptide and attached to it through surface and physical binding, then FTIR test was performed on the obtained compound. According to their results, the peaks at ~2,900 cm^−1^, and ~1,100 cm^−1^ in the GO-PEG sample indicate C-H and C-O bonds with PEG, and the peaks at ~3,280 cm^−1^, ~1,531 cm^−1^ in PGO-OH30 was related to C-O and -NH in OH30. The peak at ~815 cm^−1^ was related to the binding of OH30 peptide through π–π conjugation to GO (Mei et al., 2021). The properties of GO NSs were studied in terms of their particle size and morphology using the FE_SEM. As shown in Figures 4C,D, these nanoplates have a size between 70 nm and 400 nm, which indicates the conversion of large GO plates into GO nanosheets, and Figure 4B also shows the morphology of sharp GO NSs. Figure 4A is also the loaded image of the cyclodipeptide-rich fraction on GO NSs. According to a study conducted by Perreault et al., in 2015, GO NSs were obtained by the same method of sonication and found that the size of nanosheets has a significant effect on their antimicrobial properties. In the study they conducted, GO NSs were 0.01 μm, 0.1 μm, 0.29 μm, 0.65 μm, and the antimicrobial power increases with decreasing size (Perreault et al., 2015). Additionally, one study notes that the average size of GO NSs was approximately 32 nm with an estimated thickness of 0.8 nm (Yang et al., 2023). Therefore, the size of GO NSs can vary from tens of nanometers to micrometers depending on the study and the types of sonication methods. Table 1 shows the result of the elemental analysis of CDPs fraction loaded on GO NSs. The presence of nitrogen and the increase of carbon and oxygen confirm the peptide loading on the surface of GO NSs.

**Table 1:** EDAX analysis.

Elements	GO NSs			CDPs fraction loaded on GO NSs		
	Weight %	Atomic %	Error %	Weight %	Atomic %	Error %
C	52.16	59.22	8.83	31.90	37.61	9.16
O	47.84	40.78	15.07	51.45	45.55	12.33
N	0	0	0	16.65	16.84	22.96

### 3.3 Antibacterial activity

Standard methods were employed to test the antibacterial activity of the isolated compounds against two bacterial strains. The values for MIC and MBC were also determined and can be found in Table 2. The antimicrobial properties of CDPs are due to their preferential action on bacterial membranes compared to mammalian cells. The permeability of the membrane is increased by this, which in turn causes the collapse of ion potentials across the membrane and leads to rapid cell death. CDPs have a unique abiotic structure and act quickly against bacteria, which may limit the acquisition of drug-resistant bacteria in the long run. Low-molecular-weight CDPs have the potential to fight various existing and emerging infectious diseases (Fernandez-Lopez et al., 2001). Graphene’s antibacterial mechanism involves both physical and chemical modes of action. Physical damages are the most common and are caused by the direct contact of the sharp edge of graphene with bacterial membranes. Meanwhile, chemical modes of action are associated with oxidative stress generated by charge transfer and reactive oxygen species (ROS). This oxidative stress is the major cause of GO toxicity. The deactivation of bacterial proteins and lipids via ROS produced by the GO leads to the inability of bacteria to proliferate (Aunkor et al., 2020). The antibacterial activity of CDPs was enhanced when used together with GO NSs, leading to a significant increase in their effectiveness. In this study, the compounds showed greater antibacterial activity against Gram-positive bacteria than Gram-negative bacteria, which may be due to differences in the wall structure and the presence of two membranes in Gram-negative causes cyclodipeptides to penetrate harder due to their cyclic structure. Moreover, the tested compounds exhibited stronger antibacterial effects against standard strains compared to clinical isolates. The reason for this difference can be the presence of antibiotic resistance in clinical isolates due to the presence of efflux pumps, protease, and hydrolysis enzymes. The CDPs fraction loaded on GO NSs displayed excellent antibacterial properties, making it a promising candidate for replacing traditional antimicrobial substances.

**Table 2:** Antibacterial activities of cyclic dipeptides and GO NSs against clinical isolates and standard bacteria.

Strains		Minimum inhibitory concentration (mg/mL)			Minimum bactericidal concentration (mg/mL)			Checker board			
		MIC (CDP)	MIC (GO NSs)	MIC (CDP + GO NSs)	MBC (CDP)	MBC (GO NSs)	MBC (CDP + GO NSs)	FIC (CDP)	FIC (GO NSs)	FIC (CDP + GO NSs)	Result
Clinical isolates	*A. baumannii* 1	2.5	8	2.5	5	16	5	0.2	0.2	0.4	Synergistic
	*A. baumannii* 2	2.5	8	2.5	5	16	5	0.2	0.2	0.4	Synergistic
	*A. baumannii* 3	2.5	4	2.5	5	8	5	0.2	0.2	0.4	Synergistic
	*A. baumannii* 4	2.5	8	2.5	5	16	5	0.2	0.2	0.4	Synergistic
	*A. baumannii* 5	2.5	8	2.5	5	16	5	0.2	0.2	0.4	Synergistic
	*A. baumannii* 6	2.5	8	2.5	5	16	5	0.2	0.2	0.4	Synergistic
	*A. baumannii* 7	2.5	8	2.5	5	16	5	0.2	0.2	0.4	Synergistic
	*A. baumannii* 8	2.5	8	2.5	5	16	5	0.2	0.2	0.4	Synergistic
	*A. baumannii* 9	2.5	8	2.5	5	16	5	0.2	0.2	0.4	Synergistic
	*A. baumannii* 10	2.5	8	2.5	5	16	5	0.2	0.2	0.4	Synergistic
	*A. baumannii* 11	2.5	8	2.5	5	16	5	0.2	0.2	0.4	Synergistic
	*A. baumannii* 12	1.25	4	1.25	2.5	8	2.5	0.2	0.2	0.4	Synergistic
	*A. baumannii* 13	2.5	8	2.5	5	16	5	0.2	0.2	0.4	Synergistic
	*A. baumannii* 14	2.5	8	2.5	5	16	5	0.2	0.2	0.4	Synergistic
	*A. baumannii* 15	2.5	8	2.5	5	16	5	0.2	0.2	0.4	Synergistic
	*A. baumannii* 16	2.5	8	2.5	5	16	5	0.2	0.2	0.4	Synergistic
	*S. aureus* 1	1.25	4	1.25	2.5	8	2.5	0.2	0.2	0.4	Synergistic
	*S. aureus* 2	1.25	4	1.25	2.5	8	2.5	0.2	0.2	0.4	Synergistic
	*S. aureus* 3	1.25	4	1.25	2.5	8	2.5	0.2	0.2	0.4	Synergistic
	*S. aureus* 4	1.25	2	1.25	2.5	4	2.5	0.2	0.2	0.4	Synergistic
	*S. aureus* 5	1.25	4	1.25	2.5	8	2.5	0.2	0.2	0.4	Synergistic
Standard bacteria	*A. baumannii* ATCC19606	2.5	4	0.62	5	8	1.25	0.26	0.16	0.42	Synergistic
	*S. aureus* ATCC25923	1.25	2	0.31	2.5	4	0.62	0.25	0.16	0.41	Synergistic

### 3.4 Checkerboard assay

Table 2 shows outcomes of the *in-vitro* checkerboard interactions between CDPs and GO NSs against medically important bacteria. The FICs values were interpreted as follows: an FICs value of ≤0.5 indicated synergy, a value of 1 to 4 indicated indifference, and a value of >4 indicated antagonism (Lee et al., 2012). It has never been demonstrated before that the combination of CDPs and GO NSs has been effective against bacteria. Our study’s checkerboard assay data suggests that the combination of CDPs and GO NSs had the highest level of synergy. None of the combinations exhibited antagonism or indifference. Combining these two substances increases their antibacterial properties. Previous studies have shown that both CDPs and GO NSs have antibacterial properties. The combination of the two compounds would also affect the test bacteria through a new pathway, which could be distinct from the mechanism of action of CDPs and GO NSs, and this could be one of the reasons for the improved activity.

### 3.5 Impact of the studied compound on biofilm formation

**Figure 5:**
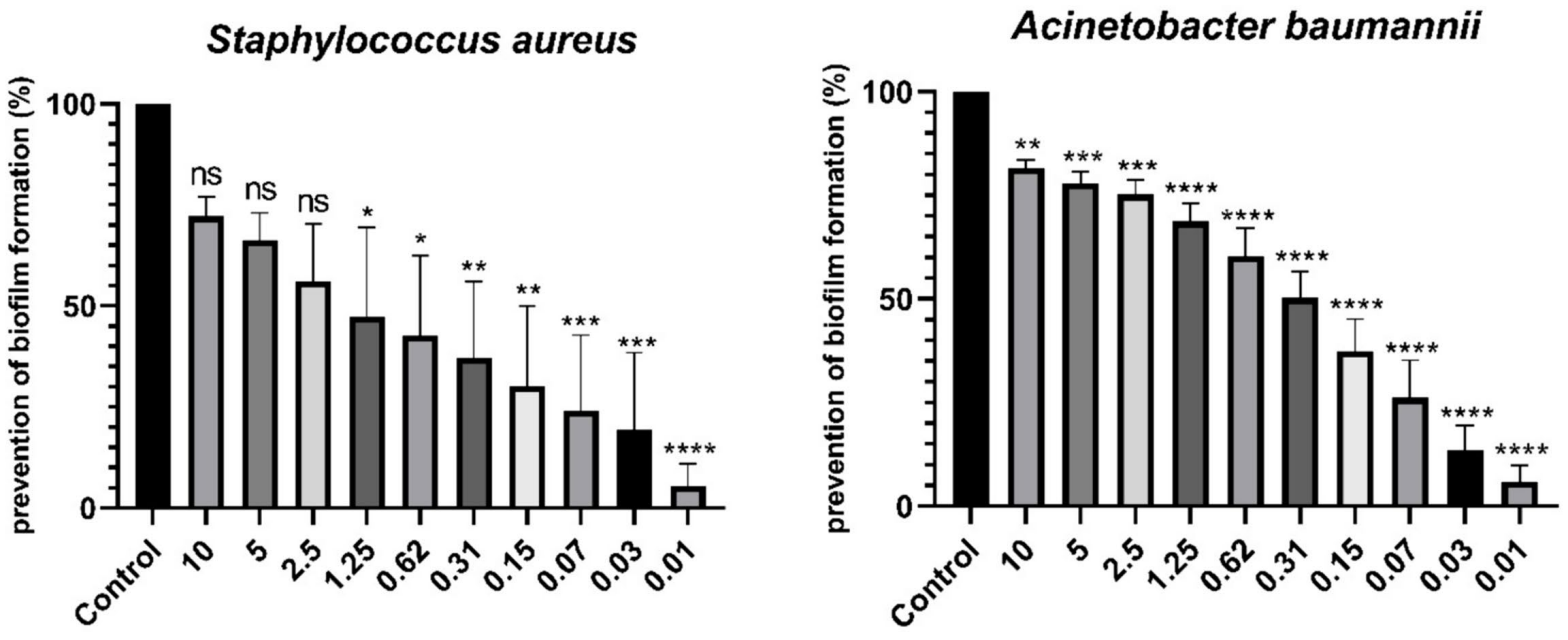
Comparison of prevention of biofilm formation (%). * Indicates a statistically significant difference (*p* < 0.05), ** indicates a statistically significant difference (*p* < 0.01), *** indicates a statistically significant difference (*p* < 0.001), and **** indicates a statistically significant difference (*p* < 0.0001). *p* values less than 0.05 with a confidence interval of 95% were considered statistically significant and ns indicates non-significant difference.

In this study, the OD_avg_ of negative control was 0.313 ± 0.016, Hence, OD_cut_ was found to be 0.361. Classification criteria for biofilms along with optical densities obtained in this study listed in Tables 3, 4. After staining the wells of the 96-well plate, it was read with an ELISA microplate reader at 595 nm. Figure 5 shows the prevention of biofilm formation in the studied bacteria. In the case of *A. baumannii*, the effect of the studied compound in all concentrations were statistically significant on the bacteria, and at a concentration of 10 mg/mL, 81.6% (*p* < 0.01) prevented the production of biofilm. In the case of *S. aureus*, the effect of the studied compound from the concentrations of 1.25 mg/mL to 0.01 mg/mL were statistically significant, and at the concentration of 1.25 mg/mL, 47.5% (*p* < 0.05) prevented the biofilm formation. In 2019, a study on the antibiofilm property of CDPs on oral pathogens was conducted by Simon et al., They found that CDPs caused reduced biofilm formation by *Streptococcus mutans* compared to control wells at concentrations of less than 6 μg/mL to 12 μg/mL (Simon et al., 2019). One of the reasons for the difference in the results was that in this article, they made these CDPs through chemical synthesis, and the bacteria studied in this study was *Streptococcus mutans*.

**Table 3:** Classification criteria for biofilms.

OD_cut_ = OD_avg_ of negative controls +3 × standard deviation (SD) of ODs of negative controls	0.361
OD ≤ OD_cut_ → Non biofilm	OD ≤ 0.361
OD_cut_ < OD ≤ 2 × OD_cut_ → Weak biofilm	0.361 < OD ≤ 0.722
2 × OD_cut_ < OD ≤ 4 × OD_cut_ → Moderate biofilm	0.722 < OD ≤ 1.444
OD > 4 × OD_cut_ → Strong biofilm	OD > 1.444

**Table 4:** Categorization of biofilm formation.

Bacteria	Categorization of biofilms	Number of bacteria
*A. baumannii* (*n* = 16)	Weak	0
	Moderate	2
	Strong	14
*S. aureus* (*n* = 5)	Weak	0
	Moderate	1
	Strong	4

## 4 Conclusion

Antibiotics contribute to the development of resistance to them. As a result, there has been increased focus on researching and implementing new antimicrobial techniques and materials. Studies have suggested that peptides created from probiotic bacteria and GO nanosheets that have antibacterial properties could be a substitute for antibiotics. Our study has found that GO NSs have antimicrobial properties and can boost the effects of CDPs against *S. aureus* and *A. baumannii*. The use of antimicrobial peptides derived from probiotic bacteria is an exciting area of study, with the potential to combat pathogenic bacteria and generate novel antimicrobial nanosheets. By completing this study and other similar investigations, we may be able to discover new and innovative ways to utilize these peptides in the fight against harmful bacteria.

## Data Availability

The datasets presented in this study can be found in online repositories. The names of the repository/repositories and accession number(s) can be found in the article/supplementary material.
